# Supplementary material for: Optimizing cardiovascular risk assessment and registration in a developing cardiovascular learning health care system: Women benefit most
Source: PLOS Digit Health. 2023 Feb 8;2(2):e0000190. doi: 10.1371/journal.pdig.0000190 (PMC9931327; doi:10.1371/journal.pdig.0000190)
Supplement: S1 Table — (DOCX) [file pdig.0000190.s001.docx]

S1 Table. Cardiovascular risk factor measurement and distributions before and after UCC-CVRM initiation

|  | Before UCC-CVRM (n = 7195) | | UCC-CVRM (n = 1904) | | Δ measured | p value |
| --- | --- | --- | --- | --- | --- | --- |
|  | Measured (%) | Distributions | Measured (%) | Distributions |  |  |
| Age, years (mean (sd)) | 100 | 58 (18) | 100 | 58 (18) | 0* | - |
| Female sex (%) | 100 | 3510 (49) | 100 | 930 (48.8) | 0* | - |
| Current smoking (%) | 76 | 1230 (23) | 94 | 210 (12) | +18% | <0.0001 |
| Physical activity norm met(%)** | n/e | n/e | 87 | 306 (19) | +87% | <0.0001 |
| BMI, kg/m2 (mean (sd)) | 57 | 26 (5) | 93 | 27 (5) | +36% | <0.0001 |
| SBP, mmHg (mean (sd))  DBP, mmHg (mean (sd)) | 77  77 | 136 (25)  78 (14) | 93  93 | 139 (23)  80 (12) | +16%  +16% | <0.0001  <0.0001 |
| Total cholesterol, mmol/L (mean (sd)  LDL-c, mmol/L (mean (sd))  HDL, mmol/L (mean (sd))  TRIGL, mmol/L (median (25-75^th^ percentile)  eGFR, min ((median (25-75^th^ percentile)  HbA1c, mmol/mol (mean (sd)) | 39  33  37  38  76  25 | 5.3 (1.6)  3.1 (1.3)  1.4 (0.4)  1.6 (1.1-2.4)  79 (60-90)  47 (18) | 84  83  83  84  87  82 | 5.2 (1.4)  3.0 (1.2)  1.4 (0.4)  1.5 (1.0-2.1)  85 (68-99)  40 (11) | +45%  +50%  +46%  +46%  +11%  +57% | <0.0001  <0.0001  <0.0001  <0.0001  <0.0001  <0.0001 |

UCC-CVRM – Utrecht Cardiovascular Cohort, BMI – Body Mass Index, SBP – Systolic Blood Pressure, DBP – Diastolic Blood Pressure, LDL-C – Low-Density-Lipoprotein cholesterol, HDL-c – High-Density-Lipoprotein cholesterol, eGFR – estimated Glomerular Filtration Rate, HbA1c – glycated hemoglobin, n/e - not extractable. *matched criterium, ** physical activity norm: ≥30 minutes of moderate activity per day(14)
