# Supplementary material for: Optimizing cardiovascular risk assessment and registration in a developing cardiovascular learning health care system: Women benefit most
Source: PLOS Digit Health. 2023 Feb 8;2(2):e0000190. doi: 10.1371/journal.pdig.0000190 (PMC9931327; doi:10.1371/journal.pdig.0000190)
Supplement: S2 Table — (DOCX) [file pdig.0000190.s002.docx]

S2: Table. Presence of risk factor measurement before and after UCC-CVRM initiation, stratified for sex

|  | Before UCC-CVRM measured (%) | | Δ measured  male | Female | | Δ measured  female |
| --- | --- | --- | --- | --- | --- | --- |
|  | Before UCC-CVRM  N = 3685  Measured (%) | UCC-CVRM  N= 974  Measured (%) |  | Before UCC-CVRM  3510  Measured (%) | UCC-CVRM  390  Measured (%) |  |
| Age | 100 | 100 | 0 | 100 | 100 | 0% |
| Current smoking | 82 | 95 | +13% | 70 | 93 | +23% |
| Physical activity norm | 0 | 87 | +87% | 0 | 87 | +87% |
| BMI | 63 | 94 | +31% | 52 | 91 | +39% |
| SBP  DBP | 82  82 | 94  94 | +12%  +12% | 72  72 | 91  91 | +19%  +19% |
| Total cholesterol  LDL-c  HDL  Triglycerides  eGFR  HbA1c | 42  35  40  41  82  28 | 81  79  80  81  87  78 | +39%  +44%  +40%  +40%  +5%  +50% | 36  32  34  35  70  22 | 87  87  87  87  87  85 | +41%  +55%  +53%  +52%  +17%  +53% |

UCC-CVRM – Utrecht Cardiovascular Cohort, BMI – Body Mass Index, SBP – Systolic Blood Pressure, DBP – Diastolic Blood Pressure, LDL-C – Low-Density-Lipoprotein cholesterol, HDL-c – High-Density-Lipoprotein cholesterol, eGFR – estimated Glomerular Filtration Rate, HbA1c – glycated hemoglobin, n/e - not extractable. * physical activity norm: ≥30 minutes of moderate activity per day(14)
