# Supplementary material for: Optimizing cardiovascular risk assessment and registration in a developing cardiovascular learning health care system: Women benefit most
Source: PLOS Digit Health. 2023 Feb 8;2(2):e0000190. doi: 10.1371/journal.pdig.0000190 (PMC9931327; doi:10.1371/journal.pdig.0000190)
Supplement: S3 Table — (DOCX) [file pdig.0000190.s003.docx]

S3: Table. Risk factor distributions before and after UCC-CVRM initiation, stratified for sex

|  | Male distributions | | Female distributions | |
| --- | --- | --- | --- | --- |
|  | Before UCC-CVRM | UCC-CVRM | Before UCC-CVRM | UCC-CVRM |
| Age, years (mean (sd) | 61 (15) | 61 (16) | 56 (19) | 56 (19) |
| Current smoking (%) | 733 (24) | 117 (13) | 497 (20) | 93 (11) |
| Physical activity norm (%)* | - | 184 (22) | - | 122 (15) |
| BMI, kg/m2 (median (25-75^th^ percentile)) | 26 (24-29) | 27 (24-29) | 26 (23-29) | 26 (22-30) |
| SBP, mmHg (mean (sd))  DBP, mmHg (mean (sd)) | 136 (23)  78 (14) | 141 (21)  81 (12) | 137 (27)  77 (15) | 136 (24)  79 (12) |
| Total cholesterol, mmol/L (median (25-75^th^ percentile))  LDL-c, mmol/L (mean (sd))  HDL, mmol/L (mean (sd))  TRIGL, mmol/L (median (25-75^th^ percentile))  eGFR, min(median (25-75^th^ percentile))  HbA1c, mmol/mol (median (25-75^th^ percentile)) | 4.9 (4.1-5.8)  3.0 (1.3)  1.2 (0.3)  1.7 (1.2-2.7)  80 (60-90)  41 (36-54) | 4.9 (4.1-5.7)  2.9 (1.3)  1.3 (0.4)  1.6 (1.1-2.3)  84 (66-98)  37F (34-43) | 5.3 (4.4-6.3)  3.2 (1.3)  1.5 (0.4)  1.3 (0.9-2.0)  77 (60-90)  39 (35-51) | 5.3 (4.4-6.2)  3.2 (1.2)  1.5 (0.4)  1.3 (0.9-2.0)  87 (70-101)  36 (33-40) |

UCC-CVRM – Utrecht Cardiovascular Cohort, BMI – Body Mass Index, SBP – Systolic Blood Pressure, DBP – Diastolic Blood Pressure, LDL-C – Low-Density-Lipoprotein cholesterol, HDL-c – High-Density-Lipoprotein cholesterol, eGFR – estimated Glomerular Filtration Rate, HbA1c – glycated hemoglobin, n/e - not extractable. * physical activity norm: ≥30 minutes of moderate activity per day
