# Supplementary material for: Optimizing cardiovascular risk assessment and registration in a developing cardiovascular learning health care system: Women benefit most
Source: PLOS Digit Health. 2023 Feb 8;2(2):e0000190. doi: 10.1371/journal.pdig.0000190 (PMC9931327; doi:10.1371/journal.pdig.0000190)
Supplement: S4 Table — (DOCX) [file pdig.0000190.s004.docx]

S4: Table: Likelihood to miss uncontrolled risk factor calculations

|  | Unmeasured % | | Uncontrolled % | | Missed  (unmeasured * uncontrolled) | | RR to miss  (missed before UCC-CVRM/missed UCC-CVRM) | Reduction missed  (100-(100*RR to miss)) |
| --- | --- | --- | --- | --- | --- | --- | --- | --- |
|  | Before UCC-CVRM | UCC-CVRM | Before UCC-CVRM | UCC-CVRM | Before UCC-CVRM | UCC-CVRM |  |  |
| SBP | 23% | 7% | 45% | 48% | 10.4% | 3.4% | 0.33 | 67% |
| LDL | 67% | 17% | 65% | 66% | 44% | 11% | 0.25 | 75% |
| HbA1c | 75% | 18% | 26% | 11% | 19.5% | 2.0% | 0.10 | 90% |
| *HbA1c* | *75%* | *23%* | *11%** | *11%* | *8.3%* | *2.0%* | *0.24* | *76%* |

* assumption before UCC-CVRM overrepresentation of high risk patients thus proportion of uncontrolled HbA1c as in UCC-CVRM is used in this calculation.
