# Supplementary material for: Optimizing cardiovascular risk assessment and registration in a developing cardiovascular learning health care system: Women benefit most
Source: PLOS Digit Health. 2023 Feb 8;2(2):e0000190. doi: 10.1371/journal.pdig.0000190 (PMC9931327; doi:10.1371/journal.pdig.0000190)
Supplement: Attachment — Submitted filename: PDIG-D-22-00238 Response to reviewers.docx [file pdig.0000190.s005.docx]

Please include the following items when submitting your revised manuscript:

*	A rebuttal letter that responds to each point raised by the editor and reviewer(s). You should upload this letter as a separate file labeled 'Response to Reviewers'.

*	A marked-up copy of your manuscript that highlights changes made to the original version. You should upload this as a separate file labeled 'Revised Manuscript with Track Changes'.

*	An unmarked version of your revised paper without tracked changes. You should upload this as a separate file labeled 'Manuscript'.

If you would like to make changes to your financial disclosure, please include your updated statement in your cover letter. Guidelines for resubmitting your figure files are available below the reviewer comments at the end of this letter.

We look forward to receiving your revised manuscript.

Kind regards,

Alistair Johnson

Section Editor

PLOS Digital Health

Journal Requirements:

1. Please review your reference list to ensure that it is complete and correct. If you have cited papers that have been retracted, please include the rationale for doing so in the manuscript text, or remove these references and replace them with relevant current references. Any changes to the reference list should be mentioned in the rebuttal letter that accompanies your revised manuscript. If you need to cite a retracted article, indicate the article's retracted status in the References list and also include a citation and full reference for the retraction notice.

*Checked and amended where necessary*

2. Please amend your detailed Financial Disclosure statement. This is published with the article. It must therefore be completed in full sentences and contain the exact wording you wish to be published.

a State the initials, alongside each funding source, of each author to receive each grant.

b. State what role the funders took in the study. If the funders had no role in your study, please state: “The funders had no role in study design, data collection and analysis, decision to publish, or preparation of the manuscript.”

c. If any authors received a salary from any of your funders, please state which authors and which funders.

*The UCC is primarily financed by the UMC Utrecht as it is care as usual. UCC as a whole, and via MLB as chair of the UCC steering committee, was supported by a grant from the Netherlands Organization for Health Research and Development (#8480-34001) to develop feedback procedures, not for salary. The funders had no role in study design, data collection and analysis, decision to publish, or preparation of the manuscript.*

If you did not receive any funding for this study, please simply state: “The authors received no specific funding for this work.”

3. Please provide separate figure files in .tif or .eps format only and remove any figures embedded in your manuscript file. Please also ensure that all files are under our size limit of 10MB.

*Separate figure files added to the submission platform and removed from the manuscript.*

4. We noticed that you used "data not shown" in the manuscript. We do not allow these references, as the PLOS data access policy requires that all data be either published with the manuscript or made available in a publicly accessible database. Please amend the supplementary material to include the referenced data or remove the references.

*We have amended the sentences stating this for it is actually not relevant to support this by a figure or table, the text provides the full information. This now reads: This is reflected in the mean number of inclusion consults per month: 50 for holiday months (May, August, September, and December) and 77 for non-holiday months in 2018.*

5. We notice that your supplementary tables are included in the manuscript file. Please remove them and upload them with the file type 'Supporting Information'. Please ensure that each Supporting Information file has a legend listed in the manuscript after the references list.

*The supplementary tables are uploaded as supporting information and removed from the manuscript. We have added a legend for the supported information after the references list.*

Additional Editor Comments (if provided):

Apologies for the delay in reviewing this manuscript. Upon receipt of the review I am recommending minor revision.

*Thank you very much for the recommendation and the opportunity to provide a rebuttal to your and the reviewers comments.*

Reviewer #1: This is an interesting manuscript that demonstrates a real-world application of the learning healthcare system (LHS) applied in the context of cardiovascular risk assessment and management. Groenhof et al. clearly state the problem definition. Notably, they present LHS as a solution to what they define as the science-to-care gap.

The authors conducted a prospective cohort study comparing the measurement of cardiovascular risk factors before and after the implementation of the UCC-CVRM, an infrastructure implemented in the EHR allowing for standardized collection of cardiovascular-related health data. To demonstrate the benefits of this infrastructure, the authors compared the proportion of measured risk factors before and after its implementation. The other outcomes measured by the authors were the proportion of patients with an indication of treatment for a given cardiovascular condition and an estimation of the proportion of patients with potentially missed severe cardiovascular-related conditions.

The results are interesting as they have shown an increase in the registration of all risk factors after implementing UCC-CVRM (see S1 Table and S2 Table). The authors extrapolate their results a little bit further by estimating the likelihood of missing uncontrolled risk factor in patients who would have otherwise required treatment (S4 Table).

I have the following comments :

Major issues

Abstract and Introduction

1) No explicit mention of similar LHS initiatives in the cardiovascular space. Notably, there seem to have other examples in the literature that have implemented LHS in this space, and it would be essential to cite and explain how your initiative differs. In the cited article of Maddox et al., multiple examples are discussed.

*Indeed there are several examples of attempts for LHS in the cardiovascular field. We have added a paragraph in the introduction to highlight this and explain what is the rationale behind our initiative which makes it differ than others. This now reads:*

*In the cardiovascular field, attempts for LHS are described by Maddox et al [1] in the domains of “science and informatics”, “patient-clinician partnerships”, “incentives”, and “development of a continuous learning culture”. Most initiatives focus on either of the domains, using for example EHR data for predictive modelling [Amarasingham et al. med care 2010; 48:981-988], using EHR data for effective cohort identification or eCRF extraction [33248277], identifying incentives that are associated with clinical improvement, using the LHS as a culture shift for continuous learning (1). The UCC as a whole was designed to be able to facilitate all domains, catalyzed by the basis of solid cardiovascular risk assessment. Furthermore, we included all patients visiting any department with a link to/dedicated to cardiovascular disease, connecting 8 departments in our hospital (and primary care) and thus creating a synergetic basis for interdisciplinary collaborations.*

Methods

2) Please add p-values to show the significance of the multiple statistical tests conducted (S1 Table, S2 Table

*p-values added*

Table 2. Cardiovascular risk factor measurement and distributions before and after UCC initiation

|  | Before UCC (n = 7195) | | UCC (n = 1904) | | Δ measured | p-value |
| --- | --- | --- | --- | --- | --- | --- |
|  | Measured (%) | Distributions | Measured (%) | Distributions |  |  |
| Age, years (mean (sd)) | 100 | 58 (18) | 100 | 58 (18) | 0* | - |
| Female sex (%) | 100 | 3510 (49) | 100 | 930 (48.8) | 0* | - |
| Current smoking (%) | 76 | 1230 (23) | 94 | 210 (12) | +18% | <0.0001 |
| Physical activity norm met(%)** | n/e | n/e | 87 | 306 (19) | +87% | <0.0001 |
| BMI, kg/m2 (mean (sd)) | 57 | 26 (5) | 93 | 27 (5) | +36% | <0.0001 |
| SBP, mmHg (mean (sd))  DBP, mmHg (mean (sd)) | 77  77 | 136 (25)  78 (14) | 93  93 | 139 (23)  80 (12) | +16%  +16% | <0.0001  <0.0001 |
| Total cholesterol, mmol/L (mean (sd)  LDL-c, mmol/L (mean (sd))  HDL, mmol/L (mean (sd))  TRIGL, mmol/L (median (25-75^th^ percentile)  eGFR, min ((median (25-75^th^ percentile)  HbA1c, mmol/mol (mean (sd)) | 39  33  37  38  76  25 | 5.3 (1.6)  3.1 (1.3)  1.4 (0.4)  1.6 (1.1-2.4)  79 (60-90)  47 (18) | 84  83  83  84  87  82 | 5.2 (1.4)  3.0 (1.2)  1.4 (0.4)  1.5 (1.0-2.1)  85 (68-99)  40 (11) | +45%  +50%  +46%  +46%  +11%  +57% | <0.0001  <0.0001  <0.0001  <0.0001  <0.0001  <0.0001  <0.0001 |

UCC – Utrecht Cardiovascular Cohort, BMI – Body Mass Index, SBP – Systolic Blood Pressure, DBP – Diastolic Blood Pressure, LDL-C – Low-Density-Lipoprotein cholesterol, HDL-c – High-Density-Lipoprotein cholesterol, eGFR – estimated Glomerular Filtration Rate, HbA1c – glycated haemoglobin, n/e - not extractable. *matched criterion, ** physical activity norm: ≥30 minutes of moderate activity per day(14)

3) I don’t agree with the calculation of the likelihood of missing hypertension: in the context of EHR documentation, not being reported does not equate not being measured. Moreover, your calculation implies a uniform distribution among unmeasured variables, which is also incorrect. I like the idea you explored with those calculations, but I would like to see a few sentenced conceding the limitation of the methodology.

*We have added a paragraph on informed missingness to the discussion to support and explore your concerns. This now reads: “*

*The limitation of using routine care data for our analysis, as for any routine care data analyses, is the restriction to what is reported in the EHR. We assume here that everything what was measured, was reported in the EHR. As we used mostly lab-based or other very much structured data (apart from smoking, which we used a validated free-text mining algorithm for [Groenhof et al]))we think that our extractions are as complete as possible. Yet, not testing – thus reporting- a specific risk factor can also be regarded as meaningful as a) the factor was considered to be normal (for example: normal BMI appearance of a patient) and thus b) not relevant to clinical decision making (no proportion attributable risk by BMI for this patient). This missing not at random (MNAR, the choice for or against a reported factor contains meaning about this factor) pattern could have made us overestimate the likelihood of missing a clinically relevant risk factor.(* Groenwold RHH. Informative missingness in electronic health record systems: the curse of knowing. *Diagnostic Progn Res*. 2020;4(1):1-6.*) Because these likelihoods are based on the assumption of equal distribution of these values among unreported values.*

Figures

4) Figure 5 and 6 do not allow for easy comparison of the pre/post CVRM implementation. I would suggest using side-by-side bar chart or use the S1 and S2 tables instead.

*We have provided the information requested in the supplement.*

Minor issues

1) Minor grammar issues and sentence structures could benefit from some touch-up.

i.e. lines 48-50 could be rewritten for simplicity «Healthcare is challenged by a growth in patient numbers, patients of higher age, patients with multiple diseases and medications, and a higher level of chronicity of disease. »

*Thank you for this suggestion. We have amended this sentence. It now reads:*

*Healthcare is challenged by a growth in patient numbers. Furthermore, these patients are of higher age, suffer from multiple diseases – including a higher portion of chronic diseases, and use more medications.*

2) Clarification of the statement line 275. Please clarify how CDSS relates or complements the work you presented in this manuscript. The last paragraph was not so clear to me.

*CDSS need complete information, for otherwise they cannot support decision making. As presented, the UCC approach enhances completeness of CVRM information thus enhances use of such systems. To clarify this argument was added to the paragraph. This now reads: …”UCC-CVRM has shown to promote optimal care, to provide feedback on the quality of care, and facilitate research. Now, sufficient quality and amount of data is available to support automated feedback processes and decision support, for these computerized decision support systems (CDSS) require complete input data.(11) A CDSS was developed to aid guideline adherent cardiovascular risk management…”*

3) Add a discussion regarding the interaction of CVRM initiation and female sex. Notably, elaborate on the potential reasons for which the results are significant for eGFR, A1c and not for SBP and smoking.

*We do not entirely understand the issue. There is an improvement in completeness of all CVRM factors on which we put effort to increase. As women, compared to men, start with a lower prevalence of available measurements, a finding that has been reported before. After UCC-CVRM initiation have a nearly equal prevalence of available measurements, it stands to reason that the ‘increase’ is larger in women than in men. So we do not understand what we need to discuss here in response to the comment of the reviewer.*
